# A Coordinated Suite of Wild-Introgression Lines in *Indica* and *Japonica* Elite Backgrounds

**DOI:** 10.3389/fpls.2020.564824

**Published:** 2020-11-12

**Authors:** Namrata Singh, Diane R. Wang, Liakat Ali, HyunJung Kim, Kazi M. Akther, Sandra E. Harrington, Ju-Won Kang, Ehsan Shakiba, Yuxin Shi, Genevieve DeClerck, Byron Meadows, Vishnu Govindaraj, Sang-Nag Ahn, Georgia C. Eizenga, Susan R. McCouch

**Affiliations:** ^1^Plant Breeding and Genetics Section, School of Integrative Plant Science, Cornell University, Ithaca, NY, United States; ^2^Rice Research and Extension Center, University of Arkansas, Stuttgart, AR, United States; ^3^Department of Agronomy, Chungnam National University, Daejeon, South Korea; ^4^USDA-ARS Dale Bumpers National Rice Research Center, Stuttgart, AR, United States

**Keywords:** *Oryza sativa*, crop wild relatives, *Oryza rufipogon* Species Complex, chromosome segment substitution line, pre-breeding resources

## Abstract

Rice, *Oryza sativa* L., is a cultivated, inbreeding species that serves as the staple food for the largest number of people on earth. It has two strongly diverged varietal groups, *Indica* and *Japonica*, which result from a combination of natural and human selection. The genetic divergence of these groups reflects the underlying population structure of their wild ancestors, and suggests that a pre-breeding strategy designed to take advantage of existing genetic, geographic and ecological substructure may provide a rational approach to the utilization of crop wild ancestors in plant improvement. Here we describe the coordinated development of six introgression libraries (*n* = 63 to 81 lines per library) in both *Indica* (cv. IR64) and *Japonica* (cv. Cybonnet) backgrounds using three bio-geographically diverse wild donors representing the *Oryza rufipogon* Species Complex from China, Laos and Indonesia. The final libraries were genotyped using an Infinium 7K rice SNP array (C7AIR) and analyzed under greenhouse conditions for several simply inherited (Mendelian) traits. These six interspecific populations can be used as individual Chromosome Segment Substitution Line libraries and, when considered together, serve as a powerful genetic resource for systematic genetic dissection of agronomic, physiological and developmental traits in rice.

## Introduction

Modern-day plant breeders seldom return to wild gene pools to rigorously explore crop wild relatives as a source of variation for plant improvement (Hajjar and Hodgkin, 2007; Warschefsky et al., 2014; Dempewolf et al., 2017). This is because using wild or exotic materials is disruptive to established breeding programs; when they are crossed to elite breeding lines, the influx of new alleles throughout the genome disturbs carefully constructed gene complexes that are the basis of highly valued traits in commercial varieties (Dempewolf et al., 2012). Even as favorable alleles are introduced, they are frequently accompanied by linked deleterious ones that require effort to eliminate. Sterility barriers may also hamper the generation of fertile offspring, adding significant time and cost to variety development (Zamir, 2001; Haussmann et al., 2004). Furthermore, it is difficult to predict the genetic potential of exotic materials because quantitatively inherited phenotypes are often masked until the accessions have been repeatedly backcrossed into well-adapted varieties (Frey et al., 1984; Eshed and Zamir, 1995; Tanksley and McCouch, 1997). Instead, for over half a century, plant breeders have been able to make reliable genetic gain in staple food crops by crossing and recombining variation within elite gene pools, fine-tuning varieties to fit target environments and relevant agricultural practices. Indeed, the combination of improvements in breeding and management has enabled steady increases in global production of staple crops such as maize (*Zea mays* L.), wheat (*Triticum aestivum* L.) and rice (*Oryza sativa* L.) for over 60 years (Food and Agriculture Organization of the United Nations, 2020).

Despite the steady results hitherto achieved with this approach, new challenges lie on the horizon. Paleoclimatic evidence suggests that we are coming to the end of an unprecedented period of global climate stability that ushered in the era of agriculture during the Pleistocene-Holocene transition (Feynman and Ruzmaikin, 2018). Increased variability in temperature and rainfall patterns are forecasted to escalate occurrences of extreme drought, flooding, wind, salt incursion, and pest and disease infestation (Rosenzweig et al., 2014; Fita et al., 2015). By 2050, an additional two billion people will share our planet (United Nations Department of Economic and Social Affairs, 2017), placing even greater pressure on plant-based food, fiber, feed and fuel production (Tilman et al., 2002; Godfray et al., 2010). These upheavals create new impetus for crop improvement programs to respond to a multitude of challenges, and new varieties must address the changing biophysical and economic constraints of growers and producers as well as the evolving demands and aspirations of consumers. Never has there been a more urgent call for plant breeders to take on so many significant challenges at once.

As the far-reaching effects of climate change are increasingly recognized and the need to reconcile short term gains in productivity with the long term viability of agricultural ecosystems becomes more apparent, plant breeders, policy makers and agriculturalists seek new ways of incorporating an expanded palette of genetic variation in farmers’ fields (Dempewolf et al., 2014; Pilling et al., 2020). There is long-standing interest in wild and exotic germplasm as a source of genetic resilience and potentially as a source of novel nutritional and/or quality traits (Redden et al., 2015; Dempewolf et al., 2017; Li et al., 2018; Burgarella et al., 2019; Rosyara et al., 2019). However, efforts to explore the range of genetic variation found in wild and exotic ancestors have been largely *ad hoc* and are undertaken without a clear path to commercial variety release. This is not to discount the many examples of successful pre-breeding, where wild alleles been shown to confer adaptive advantages in advanced breeding lines (Tanksley and McCouch, 1997; Zamir, 2001; Gur and Zamir, 2004; McCouch et al., 2007; Dwivedi et al., 2008; Mammadov et al., 2018), but rather to point out that these efforts are costly, time consuming and, in a majority of cases, fail to traverse the “last mile” to find their way into farmers’ fields. A more comprehensive understanding of how to systematically explore diverse sources of natural variation, predict genetic value and efficiently incorporate exotic variation into elite cultivated genetic backgrounds is the first step toward enhancing the value of plant genetic resources in the context of crop improvement (Warschefsky et al., 2014; Street et al., 2016; Migicovsky and Myles, 2017; Prohens et al., 2017; Li et al., 2018).

In rice, the *Oryza rufipogon* Species Complex (ORSC) is the progenitor of *O. sativa*. It is found dispersed throughout a wide range of habitats across South, Southeast and Eastern Asia (Oka, 1988; Vaughan et al., 2008). Over the last million years, the ORSC has evolved and diversified on both sides of the Himalayan Mountains, successfully adapting to highly variable ecosystems and environmental conditions (Guo and Ge, 2005; Stein et al., 2018). An evaluation of genome-wide variation in a collection of 286 ORSC accessions originating from 15 countries revealed six subpopulations reflecting both perennial and annual types of rice distributed across the geographical range of the species complex. Three of the ORSC subpopulations were found to be genetically related to the three most divergent *O. sativa* subpopulations, *Indica*, *Aus*, and *Japonica*, raising interesting questions about cross-compatibility, wild-cultivated combining ability, and shared allele complexes. To explore these questions, we undertook the development of a coordinated set of wild × cultivated chromosome segment substitution line (CSSL) libraries by systematically recombining distinct subpopulations of both wild and cultivated forms of rice.

The objective of this study was to develop and make available a suite of wild × cultivated rice CSSLs to stimulate both scientific discovery and translation in breeding, while helping to bridge the gap between genebanks and crop improvement. We provide a resource that can be rigorously evaluated in diverse environments using advanced phenotyping technologies, genetically dissected and/or expanded through crossing and/or genome manipulation, and shared widely with researchers and breeders with a common interest in exploring the genetic potential of crop wild relatives. Toward this goal, we generated six introgression libraries in elite *Indica* (cv. IR64) and *Japonica* (cv. Cybonnet) backgrounds using three genetically and bio-geographically diverse ORSC donors from China, Laos and Indonesia. This coordinated set of CSSL libraries is the first of its kind; it will enable investigators to begin to systematically examine the impact of wild introgressions in two very different genetic backgrounds, to mix and match introgressions from the same or different wild donor genomes, and to compare and contrast the phenotypic impact of introgressions in homozygous or heterozygous combinations. These materials provide the basis for predicting whether some wild subpopulations combine more productively with *Indica* versus *Japonica* varieties and offer insights about the allelic series in diverse germplasm. Deeper understanding about which combinations of wild by elite parents are most likely to give rise to superior offspring will help provide a roadmap to design pre-breeding programs that enable rice breeders to make more efficient use of wild and exotic plant genetic resources.

## Materials and Methods

### Parental Selection and Initial Crossing

A total of six libraries of inter-specific CSSLs were constructed in two *O. sativa* (*Indica* and *Japonica*) backgrounds using three wild (ORSC) donor accessions. To help select wild donors for CSSL development, genetic relationships were examined among 286 ORSC accessions and 54 *O. sativa* varieties initially using 50 Simple Sequence Repeat (SSR) markers and subsequently using a set of 113,739 GBS SNPs (Kim et al., 2016). A rooted Neighbor Joining (NJ) dendrogram using an *Oryza officinalis*, Wall ex Watt accession set as the outgroup was constructed with 100 bootstrap replicates (Tamura and Nei, 1993) in Geneious v10.0.9. ORSC donors were selected to represent geographical, phenotypic and genomic diversity, and donor selection was also influenced by cross compatibility with the cultivated recurrent parents (RPs). Three donor plants per wild accession were grown out for crossing with multiple individuals from the two RPs in the Guterman Greenhouse at Cornell University (Kim et al., 2016). Efforts to develop purified donor lines from the ORSC individuals used for crossing were not successful, and therefore additional plants were grown from the original donor seed stocks and used as proxies in some of the subsequent analyses.

### CSSL Development

Three criteria were used to select lines for generation advance over the course of development: (1) presence of a wild introgression in a target region, i.e., a region that contributed to the goal of providing coverage of the entire donor genome with independent, overlapping substituted segments, (2) minimum number of wild introgressions in non-target regions, contributing to the goal of creating a library consisting of a set of near isogenic lines, each carrying a single wild introgression in an RP genetic background and (3) recovery of a “lost introgression,” in cases where a heterozygous donor segment present in one generation was not recovered in the subsequent generation, requiring the retrieval of a line from an earlier generation. The breeding scheme is presented in Supplementary Figure S1.

Marker-assisted selection (MAS) was carried out at every generation. Foreground selection was performed in the first three generations using 384 Oligo Pool Assays (OPAs) to establish sets of lines that covered the donor genome (Thomson et al., 2012). Subsequent generations utilized 6 K or 7 K SNP arrays (the C6AIR and C7AIR; Thomson et al., 2017; Morales et al., 2020), to eliminate non-target introgressions (negative selection) and increase the proportion of recurrent parent background. Length of each donor segment was determined based on the physical position of the marker genotypes. For a given introgression line, the preferred range for a target donor introgression was 4–5 Mb (13–17 cM), with maximum recurrent parent background. Each introgression line was selected such that there was a 1–2 Mb overlap within its neighboring introgression line on both sides of the introgressed segment. Donor segment selection was given preference over background recovery. Therefore, if donor segments were lost during backcrossing, plants from an earlier generation with less recurrent parent recovery were selected for the final library. For each population, the set of lines representing the final library were analyzed using CSSL Finder v.0.9.722^1^. The target segment size was calculated in Microsoft Excel, whereas the number of donor and background segments, and the percent recurrent parent genome, were calculated using CSSL finder. The libraries were visualized using the CSSL finder or GGT (van Berloo, 2008) software during population development.

### Genotyping

Genomic DNA was extracted from leaf tissue from individual plants in each backcross generation using a modified CTAB protocol (Dellaporta et al., 1983; Fulton et al., 1995). As new technology became available, various genotyping methods were employed for MAS during library development. These platforms were SSRs, 384-OPA, the Cornell_6K_Array_Infinium_Rice (C6AIR) and the C7AIR (McCouch et al., 2002; Orjuela et al., 2010; Thomson et al., 2012, 2017; Morales et al., 2020). Details on each of these genotyping methods are provided in Supplementary Data 1. Genotyping of the final libraries was carried out using the C7AIR SNP array (Morales et al., 2020). The “ACGT” nucleotide file from Genome Studio was exported in the PLINK format and uploaded on TASSEL GUI^2^. The taxa (samples) were divided into six groups, each representing one of the six CSSL populations. In each library, SNPs were called based on major vs. minor allele, where it was assumed that the minor allele was inherited from the wild donor parent. SNPs with ≥39% missing data or heterozygous calls, monomorphic SNPs, and singleton SNPs were removed from each dataset. Specific SNPs were removed when it was known that one of the parents could carry either the major or the minor allele at that variant due to heterogeneity of the parental accessions. In addition to genome-wide assays, all five parental accessions in addition to CSSLs that had red or red/brown pericarp were analyzed using functional InDel markers for *Rc* developed by Sweeney et al. (2006) and sequencing primers for *Rd* described by Furukawa et al. (2007). Likewise, donors and RPs were sequenced at black hull genes, *Ph* and *BH4* using primers described in Yu et al. (2008) and Zhu et al. (2011).

### Phenotyping

Morphological variation for 16 traits was documented for donor accessions and RPs based on observations of two to five plants per accession. Parent plants were grown in 4 inch pots in the Guterman greenhouse at Cornell University (Ithaca, NY, United States) during summer 2015 and evaluated as described in Supplementary Table S1. The phenol reaction of the grain was determined on the parents following the method of Chen et al. (2014). CSSLs were also evaluated for pericarp color, hull color, seed shattering, seed set, and delayed flowering (noted as very late flowering under long (∼15 h) days.

### CSSL Statistics

Custom R scripts^3^ were written to calculate per-library and per-line statistics and to make figures of marker distribution and graphical genotypes. Percent donor genome, percent heterozygosity, and percent missingness were computed based on the total number of informative markers per library. To map QTL in the interspecific CSSL population, stepwise regression within IciMapping v4.0 software^4^ (RSTEP-LRT option) can be implemented as described in Wang et al. (2017) and Balakrishnan et al. (2020).

### Germplasm Availability

Seeds from the CSSLs and the RPs, IR64 and Cybonnet, are available from the Genetics Stocks-*Oryza* (GSOR) center in Stuttgart, AR, United States^5^. As is standard practice, five seeds per genetic stock will be provided for researchers to amplify. For partially sterile and low-yielding CSSLs, seeds from sibling or progeny lines may be provided in lieu of the original CSSL seed. Seeds from genebank accessions of the three ORSC donors are available as follows: OrA (W1944) from the National Institute of Genetics, Japan; OrB (IRGC106148) and OrC (IRGC105567) from the International Rice Germplasm Collection in the Philippines.

## Results and Discussion

### Selection of Wild and Cultivated Parents

**FIGURE 1:**
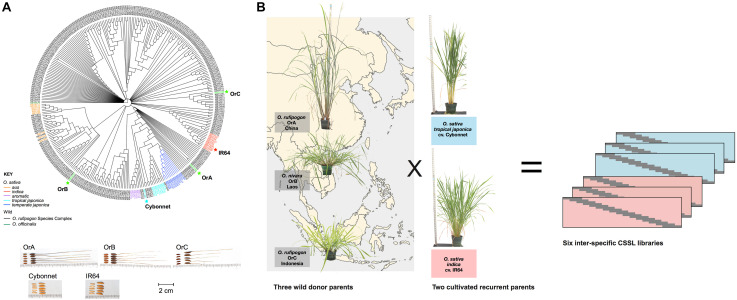
Parental selection of six inter-specific Chromosome Segment Substitution Line (CSSL) libraries. Six libraries of CSSL libraries were constructed in two elite *O. sativa* backgrounds (cv. Cybonnet and IR64) using three wild *Oryza rufipogon* species complex (ORSC) donor accessions (OrA, OrB, and OrC). Seed morphology and genetic relationships of parental lines with each other and other wild and cultivated accessions are shown in panel **(A)**. Geographical origin and plant architecture variation of the wild donors are shown in panel **(B)**.

Genetic relationships among a collection of 286 accessions from the ORSC and a set of 54 *O. sativa* control samples were examined using a rooted NJ dendrogram and a previously generated set of 113,739 GBS SNPs (Kim et al., 2016; Figure 1). Based on geographical, phenotypic and genomic diversity, as well as cross-compatibility, three ORSC accessions were selected for crossing with both *Indica* and *Japonica* elite varieties. They were (1) NIAS W1944 (hereafter referred to as OrA) from China, classified in the Institute of Genetics in Japan as *O. rufipogon* Griff.; (2) IRGC106148 (hereafter referred to as OrB) from Laos, identified as *Oryza nivara* Sharma and Shastry; and (3) IRGC105567 (hereafter referred to as OrC) from Indonesia, classified as *O. rufipogon* Griff (Table 1). These accessions represented wild subpopulations W6, W4/W1 admix, and W1, respectively, as reported by Kim et al. (2016). Genetically, this suggests that OrA is more closely related to the *Japonica* varietal group, OrB shares ancestry with *Aus* and *Indica*, and OrC represents a group of ancestors that do not share close ancestry with any *O. sativa* subpopulation.

**TABLE 1:** Parents used to construct six CSSL libraries.

Parent	Gene bank ID^(1)^	Name	Species^(2)^	Country	Nuclear genome^(3)^	Subpopulation^(4)^	Chloroplast haplotype^(3)^
Donor	NIAS W1944	OrA	*O. rufipogon*	China	W6	–	I-1
	IRGC 106148	OrB	*O. nivara*	Laos	W4/W1	–	I-1
	IRGC 105567	OrC	*O. rufipogon*	Indonesia	W1	–	VIII-2
Recurrent	PI 636726	Cybonnet	*O. sativa*	United States	–	*tropical japonica*	In-12
	IRGC 117268	IR64	*O. sativa*	Philippines	–	*indica*	I-5

*^(1)^IRGC = International Rice Germplasm Collection, IRRI, Philippines; NIAS = National Institute of Genetics, Mishima, Japan distributed by National BioResource Project: Rice; PI-Plant Introduction, distributed by the National Small Grains Collection (NSGC) at Aberdeen, ID, United States, a component of the National Plant Germplasm System (NPGS) of the United States Department of Agriculture - Agricultural Research Service.*

*^(2)^Species designation as presented in IRGC/NIAS/NSGC.*

*^(3)^As per Kim et al. (2016). W6 is *Japonica*-like; W4/W1 is *Indica* admix (*aus/indica*); W1 did not share close ancestry with any *O. sativa* subpopulation.*

Recurrent parents were selected to represent both the *Japonica* and the *Indica* varietal groups, with emphasis on elite, publicly available breeding material commonly used in public-sector breeding programs and widely grown in different parts of the world. Cultivars Cybonnet (CV-122, PI636726, GSOR301380) and IR64 (IRGC117268, GSOR301401) were selected as the RP (Table 1). Cybonnet is an elite *tropical japonica* from the United States adapted to the sub-tropics, and was released by the Arkansas Agricultural Experiment Station in 2004. It is early maturing, high-yielding, and semi-dwarf, with long-grain, improved milling yield and good blast resistance (Gibbons et al., 2006). IR64 is an elite *Indica* developed by the International Rice Research Institute (IRRI) in the Philippines and released in 1985. It is a semi-dwarf variety with high yield potential, short growth duration, enhanced resistance to diseases and insect pests and is well-known for its superior eating quality (Khush, 2005; Mackill and Khush, 2018). IR64 is adapted to the tropics and is widely grown throughout South and SE Asia. It is still commonly used as a parent for breeding of both inbred and hybrid varieties (Virmani and Kumar, 2004; Toriyama and Kazama, 2016).

### Phenotypic Diversity of Donor and Recurrent Parents

The wild donors and the elite RPs were evaluated in the greenhouse for 16 characters, with emphasis on plant and seed morphological traits, flowering time, and seed set (Table 2 and Supplementary Table S1). OrA from China was readily differentiated from the other two donors with its tall, upright plant type, compact panicles, lack of shattering, lack of stolons and elbows, as well as its early flowering. OrB from Laos had short stature, abundant stolons and elbows, spreading culm habit, open panicle type and abundant shattering, with intermediate flowering time (Figure 1). OrC from Indonesia was also short, flowered later than the other donors, had open panicles that shattered readily, and very poor seed set. All donors had black hull, long awns and positive phenol reaction. Conversely, neither recurrent parent displayed awns, elbows or a shattering habit during any greenhouse or field observations, and both had a high rate of seed production. Cybonnet had erect culm angle, compact panicles and straw colored grains. IR64 had erect culm angle with intermediate panicle type and golden hull color. Cybonnet seeds showed no reaction to phenol while IR64 seeds had a positive phenol reaction whereby seeds turned a dark purple-black color when exposed to phenol.

**TABLE 2:** List of traits evaluated under greenhouse conditions in the CSSL donors and recurrent parents.

Trait name	ORSC^a^ donor accessions			*O. sativa* recurrent accessions	
		
	OrA	OrB	OrC	Cybonnet	IR64
Culm angle	Erect (5–10°)	Spreading (>60–80°)	Open (∼60°)	Erect (4–8°)	Erect (12–17°)
Awn presence*	9, Long and fully awned	9, Long and fully awned	9, Long and fully awned	0, Absent	0, Absent
Panicle type*	1, Compact	3, Open	3, Open	1, Compact	2, Intermediate
Rhizome/stolon formation*	1, Vegetative crown	3, Vegetative crown and weak rhizomes	3, Vegetative crown and weak rhizomes	1, Vegetative crown	1, Vegetative crown
Elbows*	1, Absent	2, Present	2, Present	1, Absent	1, Absent
Plant height	avg. 197 cm	avg. 89.1 cm	avg. 99 cm	avg. 116 cm	avg. 98 cm
Days to flowering in Ithaca, NY (∼15 h daylength)	avg. 89 days	avg. 110 days	avg. 135 days	avg. 85 days	avg. 94 days
Seed production	Moderate	Moderate	Low	High	High
Seed shattering or hilum abscission*	0	9 (>50%)	9 (>50%)	0	0
Lemma and Palea: Hull color*	8, Black	8, Black	8, Black	2, Straw	3, Gold
Caryopsis: Grain color*	5, Brown	5, Red	5, Red	2, Light brown	2, Light brown
Hull length or grain length (mm)	8.3	7.3	8.4	9.3	9.8
Hull width or grain width (mm)	2.6	2.3	2	2.5	2.7
Grain length or caryopsis length (mm)	6.4	5.4	5.9	7.1	7.2
Grain width or caryopsis width (mm)	2.1	1.9	1.7	2.1	2.2
Phenol reaction^b^	1, Positive	1, Positive	1, Positive	0, Negative	1, Positive

*Numbers refer to the Standard Evaluation System (SES) for rice; see also Supplementary Table S1 for descriptions.

*^*a*^*Oryza rufipogon* Species Complex (ORSC).*

*^*b*^Determined using the method of Chen et al. (2014).*

### Characterization of CSSL Libraries

**FIGURE 2:**
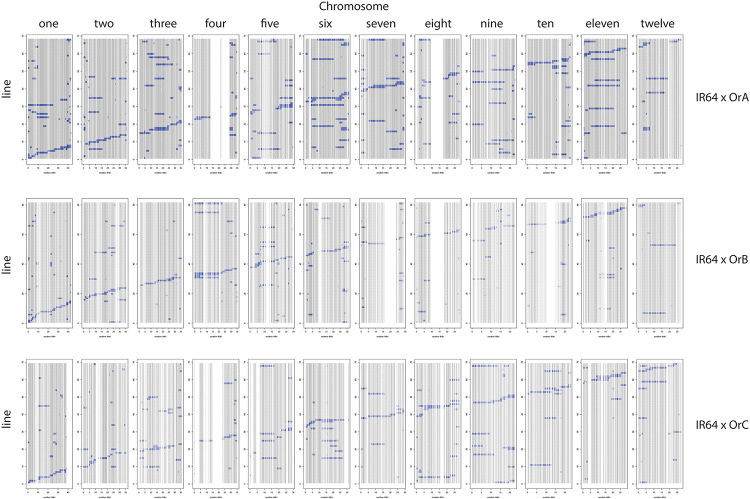
IR64 CSSL libraries using OrA (top), OrB (middle), and OrC (bottom) donors. The 12 chromosomes are identified along the top and the markers for each of the 12 chromosomes are represented as a “column” based on physical distance. The genotype of each CSSL (line) is represented as a “row” within the given library identified on the left side.

**FIGURE 3:**
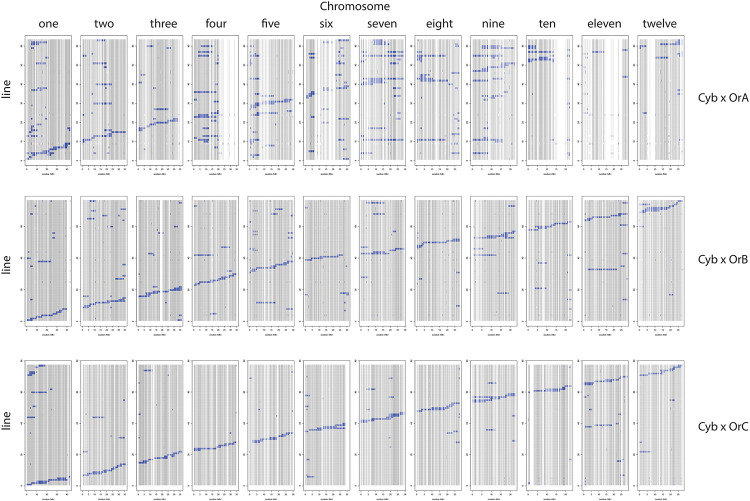
Cybonnet CSSL libraries using OrA (top), OrB (middle), and OrC (bottom) donors. The 12 chromosomes are identified along the top and the markers for each of the 12 chromosomes are represented as a “column” based on physical distance. The genotype of each CSSL (line) is represented as a “row” within the given library identified on the left side.

Table 3 provides a summary of the six CSSL libraries in terms of number of lines, donor contribution, marker coverage, and percent heterozygosity. Individual libraries are comprised of 63–81 lines each, with an estimated percent donor contribution per line ranging from 2.9 to 6.9%, based on the total number of informative markers per library. Libraries constructed using OrA gave rise to populations with the largest number of spurious background introgressions per line compared to libraries constructed using OrB or OrC, though the reason for this is not clear (Figures 2, 3).

**TABLE 3:** Summary statistics on CSSL libraries.

Recurrent parent	Cybonnet			IR64		
		
Donor parent	OrA	OrB	OrC	OrA	OrB	OrC
Number of lines	63	76	77	68	81	69
Average donor contribution* (%)	6.9	3.3	2.9	6.1	3.3	4.1
Number of informative SNPs	2116	2693	2521	2962	1496	1558
Average inter-marker distance (kb)	172	137	145	124	245	233
Average missingness* (%)	0.32	0.16	0.06	0.12	0.03	0.06
Average heterozygosity* (%)	0.99	0.46	0.17	0.73	0.17	0.22

*Average donor contribution, heterozygosity and missingness metrics are computed based on the number of markers (not physical distances or positions).

**FIGURE 4:**
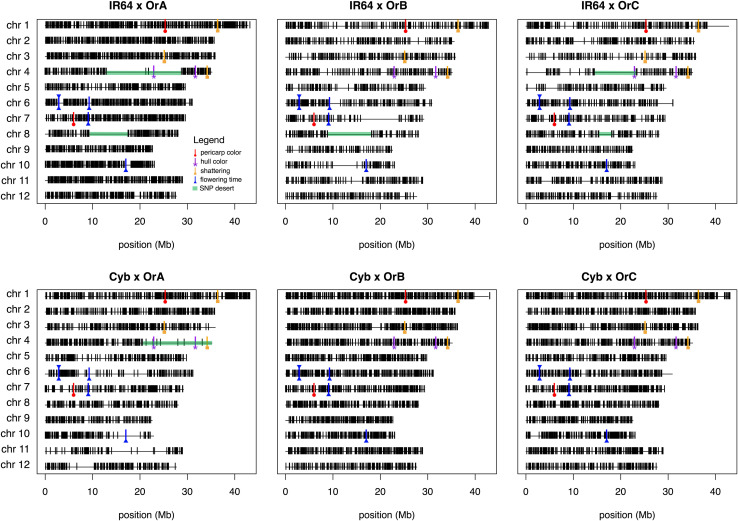
Physical distribution of markers using the C7AIR across the six finalized CSSL libraries. Total number of polymorphic markers in the populations ranged from 1496 (IR64 × OrB) to 2962 (IR64 × OrA). Visual gaps in marker distribution are due to either lack of polymorphism or poor SNP calling for which markers were filtered. Genes discussed in this manuscript are annotated using symbols shown in the legend: red line with circle for pericarp color genes (*Rc*, *Rd*); purple line with asterisk for hull color genes (*Ph*, *BH4*); orange line with square for shattering genes (*SH4*, *qSH1*, *Sh1*); and blue line with triangle flowering time genes (*RFT*, *Hd1*, *Hd3a*, *Ehd1*, *Ghd7*). Also indicated are marker gaps in green highlight that are discussed in the text.

The number of informative SNPs per population ranged from 1,496 to 2,962, and was correlated with the degree of genetic relatedness between the parents. Thus, in the Cybonnet × OrA library, the number of SNPs detected was lower than in the Cybonnet × OrB or OrC libraries, and the reverse was true for the IR64 libraries. This was consistent with the ORSC subpopulation relationships reported by Kim et al. (2016) and with the phylogenetic analysis summarized in Figure 1. The average inter-marker distance is negatively correlated with marker number, but does not adequately portray the patterns of marker distribution observed in the different libraries; these are summarized in Figure 4. The large gaps in marker coverage are most pronounced in the IR64 populations (with maximum sizes of 8.3, 9.3, and 8.8 Mb in the OrA, OrB, and OrC populations, respectively), but are also apparent in the Cybonnet populations (with maximum sizes of 4.8, 3.3, and 1.9 Mb in the OrA, OrB, and OrC populations, respectively) (regions highlighted in green in Figure 4). On chromosome 4, a marker gap at 12,954,360–21,271,163 bp in the IR64 × OrA population was found to be partially shared with a gap at 14,482,427–23,294,656 bp in the IR64 × OrC and also with a gap from 20,352,551 bp until the end of the chromosome at 35,502,694 bp in the Cybonnet × OrA population. Other examples include the gaps on chromosome 8 observed from 9,289,473 to 17,516,403 bp in IR64 × OrA, 8,897,653–18,185,078 bp in IR64 × OrB, and 15,439,243–18,108,496 bp in IR64 × OrC. These regions that are devoid of informative markers are referred to as “SNP deserts,” and are not believed to be the result of major bias in the SNP assays, based on the fact that marker coverage in the Cybonnet × OrB and Cybonnet × OrC populations is fairly uniform and that apparent gaps in one population are invariably well covered regions in another. They are indicated as white spaces in the CSSL maps in Figures 2, 3. Overall, the amount of missing data after SNP filtering was very low, ranging from 0.03 to 0.32%, and the level of heterozygosity is <1% in all libraries.

We hypothesize that the “SNP deserts” may be due to one or more of the following: (1) these regions are common by state (cbs) and inferred to be common by descent (cbd) in the different cross combinations, thereby resulting in swaths of monomorphic SNPs; (2) sterility challenges led to irrecoverable introgressions, whereby target donor segments in one generation could not be propagated into future generations, resulting in an apparent lack of polymorphism in these regions in the final library dataset; and/or (3) heterogeneity in recurrent parent accessions used for crossing gave rise to polymorphic regions in final libraries that were difficult to attribute to either donor or recurrent parent and thus markers in these regions were dropped during SNP filtering (see section “Materials and Methods”). It is difficult to explicitly attribute marker gaps to cbd (hypothesis one) because the exact individuals used for crossing may not have been the same ones used for genotyping; however, given our current understanding of the genetic relationships between wild and cultivated rice subpopulations, we postulate that it is unlikely the gap on chromosome 4 shared between IR64 × OrA, IR64 × OrC, and Cybonnet × OrA libraries is a result of cbd. As an example of hypothesis two, we report efforts to maintain a line containing an introgression covering the region on chromosome 11 from 14 to 25.7 Mb in the Cybonnet × OrA population. The line was eventually lost due to sterility, and its absence from the final library led to apparent monomorphism in the genotyping dataset, explaining the marker gap in this region for that library (Figure 4). As for hypothesis three, within-line genetic heterogeneity is common in many improved varieties of rice, and is an essential characteristic of landrace and wild accessions found in genebanks (Olufowote et al., 1997). In this study, three different individuals per wild accession were used as pollen donors in crosses with multiple individuals from the two RPs. Regions in the genome where heterogenity is prevalent among individuals of an accession pose difficulties downstream in determining whether donor introgressions are present. Future attempts to carry out deep sequencing on these lines may likewise run into similar challenges in attributing alleles to donor or recurrent parent.

### Wild Phenotypes Displayed in CSSL Lines

Depending on the specific donor segments carried by each of the CSSLs, wild characteristics are sometimes observed in the final lines. Classic domestication traits that help differentiate wild and cultivated rice include pericarp color, hull color, degree of seed shattering and flowering time. Some of these characteristics make the CSSLs difficult to grow and/or subject them to regulatory oversight, and thus knowing which lines carry “wild” traits is helpful in designing experiments to further evaluate the lines.

#### Pericarp Color

Out of the total 434 CSSLs comprising the six introgression libraries, 16 had pericarp color typically associated with wild rice (i.e., red or red/brown) (Supplementary Figure S2); 10 of these were lines in the Cybonnet background, and six were in the IR64 background. Five of the six libraries presented ILs with colored pericarp; the Cybonnet × OrC library was comprised only of white pericarp lines. Pericarp color is dependent upon the combination of alleles at the *Rc* (Os07g0211500) and *Rd* (Os01g0633500) loci, located on rice chromosomes 7 and 1, respectively (Table 4 and Figure 4). Both *Rc* and *Rd* must carry a dominant allele to produce red pericarp, whereas a dominant *Rc* allele and a recessive *rd* allele will produce brown pericarp. Any other combination (*rc/rd*, *rc/Rd*) will produce rice with white pericarp. All three donor accessions in these libraries had red pericarp and both RPs had white pericarp (Supplementary Figure S2). PCR results showed that both cultivated parents were homozygous recessive at the *Rc* locus; both carried the 14 bp deletion in exon 2 associated with lack of function for *Rc*, while all three wild parents carried the dominant (functional) allele at *Rc*. We next examined the *Rd* gene and found that Cybonnet carried the recessive (non-functional) allele but IR64 had the dominant *Rd* allele. OrB and OrC both carried the dominant (functional) allele at the *Rd* locus, but OrA was heterogeneous; some individuals carried the dominant allele and had red seeds, and others had brown seeds due to a recessive allele at *Rd*.

**TABLE 4:** Gene reference Table with fsnps added.

Gene_ID	Gene symbol synonym(s)	CGSNL Gene name	Gene name synonym(s)	Chr. No.	RAP ID	RAPDB start (bp)	RAPDB end (bp)	MSU ID
*Rc*	*RC, OsbHLH017, OsbHLH17*	*BROWN PERICARP AND SEED COAT*	*Brown pericarp and seed coat, basic helix loop helix 17*	7	Os07g0211500	6,062,889	6,069,317	LOC_Os07g11020.1
*Rd*	*RD, DFR, OsDFR, OS-DFR*	*RED PERICARP AND SEED COAT*	*Red pericarp and seed coat, dihydroflavonol 4-reductase, dihydroflavonol-4-reductase*	1	Os01g0633500	25,382,714	25,384,678	LOC_Os01g44260.1
*Bh4*	*Bh4, OsATL14, ATL14*	*BLACK HULL 4*	*Black hull 4, amino acid transporter-like 14*	4	Os04g0460200	22,969,845	22,971,859	LOC_Os04g38670.1; LOC_Os04g38660.1
*Ph*	*BHC, Bhc(Po), Bhc, Po, Ph, Bh3, PPO, Phr1, BH1*	*BLACK HULL C*	*Phenol staining, polyphenol oxidase, PPO enzyme, BLACK HULL1*	4	Os04g0624500	31,749,141	31,751,604	LOC_Os04g53300.1
*OsbHLH016*	*OsbHLH016, Kala4, OsB2*		*Basic helix-loop-helix protein 016 Key gene for black coloration by anthocyanin accumulation on chromosome 4*		Os04g0557500	27,915,598	27,939,357	LOC_Os04g47059.1
*SH4*	*Sh4, SHAT2, SHA1, qSH4, OsSh4*,	*SHATTERING 4*	*Shattering 4, grain shattering quantitative trait locus on chromosome 4, SHATTERING ABORTION2*	4	Os04g0670900	34,231,186	34,233,221	LOC_Os04g57530.1
*qSH1*	*qSH1, qsh1, Qsh1, qSH-1, RIL1, OsRIL1*	*Shattering (QTL)-1*	*QTL of seed shattering on chromosome 1, RI-LIKE1, erticillate rachis-like 1*	1	Os01g0848400	36,445,456	36,449,951	LOC_Os01g62920.1
*OsSh1*	*YAB2, OsYAB2, OsSh1, Sh1, SH1, OsFIL2, OsSH1, Osh1, qSH3*	*SHATTERING1*	*YABBY2, Os YABBY2, Shattering1, FIL homolog 2*	3	Os03g0650000	25,197,057	25,206,948	LOC_Os03g44710.1
*RFT1*	*FT-L 3, OsFTL3, FT-L3, RFT, OsRFT1*	*RICE FLOWERING- LOCUS T 1*	*Rice Flowering-locus T 1, FT-like gene 3, RFT*	6	Os06g0157500	2,926,823	2,928,474	LOC_Os06g06300.1
*Hd3a*	HD3A, FT, OsHd3a, qHD3(t)*, FT-L 2, OsFTL2	*HEADING DATE 3A*	*Heading date-3a, Heading date (QTL)-3(t), Flowering locus T, FT-like gene 2*	6	Os06g0157700	2,940,004	2,942,452	LOC_Os06g06320.1
*Hd1*	*Se1, Se1(Lm,Lf,Rs,Fl), HD1, K, Se-1, Hd1(t), qHD1(t), OsA, OsBBX18, BBX18, Hd1/OsA, OsCCT21*	*PHOTOSENSITIVITY 1*	*Photosensitivity1, Heading date, HEADING DATE 1, Arabidopsis CONSTANS(CO) gene ortholog, B-box- containing protein 18, CCT domain-containing gene 21, CCT (CO, CO-LIKE and TOC1) domain protein 21*	6	Os06g0275000	9,336,376	9,338,569	LOC_Os06g16370.1
*Ghd7*	*Ghd2, OsCCT26, OsCMF8, OsI, OsEH7 Ghd7/Hd4, EH7-1/Hd4, Ghd7-0a*,	*HEADING DATE 7*	*heading date 7, “\Grain number, plant height, and heading date7\,” CCT domain-containing gene 26, CCT (CO, CO-LIKE and TOC1) domain protein 26, Early heading 7*	7	Os07g0261200	9,152,377	9,155,030	LOC_Os07g15770.1
*Ehd1*	Ef1, Ef2, Ehd1, Eh1(t)*, qEHD-10- 1(t) (qEhd1), OsRR30, OsRR22	*EARLINESS 1*	*Earliness1, EARLY HEADING DATE 1*	10	Os10g0463400	17,076,098	17,081,344	LOC_Os10g32600.1
*Pi-ta*	*PITA, Pita (sl, Pi4a, Pi-ta), Pita2, Pi4a, Pi-4, OsTRXh6, OsTrx30*	*PYRICULARIA ORYZAE RESISTANCE TA*	*Pyricularia oryzae resistance-ta, Magnaporthe grisea resistance-ta, Blast resistance ta, Thioredoxin H-type 6, Thioredoxin 30*	12	Os12g0281300	10,606,359	10,611,917	LOC_Os12g18360.2 LOC_Os12g18360.1
*GS3*	*LK3, lk3(t), SG3, SG3-GS3, OsGS3, OsGW3, RGG3, GGC1 Mi, OsGGC1*,	*LONG KERNEL 3*	*long kernel 3, grain size, grain length and weight protein, GRAIN SIZE 3, G gamma subunit GS3, Heterotrimeric G Protein gamma Subunit*	3	Os03g0407400	16,729,501	16,735,109	Not annotated
*ALK*	*alk, OsSSIIa, SSIIa, SSS2A, SS2a, OsSSII-3*	*ALKALI DEGENERATION*	*ALKALI DEGENERATION, Soluble starch synthase 2–3, chloroplast precursor, Soluble starch synthase 2–3, chloroplastic/amyloplastic, Soluble starch synthase II- 3, Starch synthase IIa, soluble starch synthase Iia*	6	Os06g0229800	6,748,398	6,753,302	LOC_Os06g12450.1
*Waxy*	*WX1, Wx, WX-B, OsWx, GBSS, OsGBSSI, GBSS1, GSS*	*GLUTINOUS ENDOSPERM*	*glutinous endosperm, WAXY, Granule-bound starch synthase 1/chloroplastic/amyloplastic, Granule- bound starch synthase I, UDP-glycogen synthase, Granule-bound starch synthase/chloroplast precursor, glycogen [starch] synthase, UDPG-glycogen transglucosylase, uridine diphosphoglucose-glycogen glucosyltransferase*	6	Os06g0133000	1,765,622	1,770,653	LOC_Os06g04200.4 LOC_Os06g04200.3 LOC_Os06g04200.2 LOC_Os06g04200.1

Supplementary Figure S2 shows CSSLs that have donor introgressions at the *Rc* and/or *Rd* genes, and indicates the pericarp color observed in the grain. Of the 16 CSSLs that carried pigmented pericarp, eight were from the Cyb × OrA library, and none were from the Cyb × OrC library. The lines were genotyped using the methods described above and the observed pericarp color was consistent with genetic predictions in the libraries derived from OrB and OrC in both the Cybonnet and the IR64 backgrounds. On the other hand, sequencing of the *Rd* gene in the CSSLs derived from OrA revealed two separate single nucleotide substitutions (mut#1 = A and mut#2 = G) in exon 2, each of which have previously been shown to render the gene non-functional (Sun et al., 2018). In the Cyb × OrA library, some CSSLs carried the wild type allele at *Rd* while others carried a non-functional *rd* allele; all lines with a non-functional allele carried mut#2 in exon2, while a few also carried mut#1. We observed a single line, Cyb_ OrA_4, with brown pericarp due to the fact that it carried a donor introgression at *Rc* on chromosome 7 and a second donor introgression on chromosome 1 carrying the *rd* allele with both mut#1 and mut#2. Other lines (Cyb_OrA_6, IR64_OrA_5) carrying the same donor allele at *rd* (with the two mutations) had white pericarp because they carried *rc* alleles from their respective RPs on chromosome 7 (Supplementary Figure S2).

#### Hull Color

The black hull phenotype is controlled by at least two complementary genes that are linked in a cluster of domestication-related genes on rice chromosome 4 (Fukuda et al., 2012). The gene Black Hull 4 (BH4) encodes a tyrosine transporter (Zhu et al., 2011), and Phenol Reaction (Ph) encodes the polyphenol oxidase (PPO) enzyme which uses tyrosine as a substrate (Yu et al., 2008; Table 4 and Figure 4). A third gene identified as OsbHLH016 is located between the other two genes on chromosome 4, and encodes a bHLH transcription factor. It is known to positively regulate the biosynthesis of anthocyanin and has been tentatively associated with black hull (Sun et al., 2018). Other genes for black hull have been reported on chromosomes 4, 5, and 7 (Morishima and Oka, 1981; Maekawa, 1984; Gu et al., 2005). Functional alleles at both BH4 and Ph are necessary to generate the black hull phenotype, but straw-white hull color has been selected under domestication. Multiple functional mutations have been documented in both genes, with clear divergence between the *Indica* and *Japonica* gene pools, resulting in straw-gold hull color in a majority of cultivated germplasm (Yu et al., 2008; Zhu et al., 2011; Fukuda et al., 2012).

In this study, the three donor parents all had black hulls, while both RPs had straw-gold hulls (Figure 1 and Table 2). To confirm which of the parental lines carried a functional *Ph* gene, seeds of the donors and RPs were tested for phenol reaction. All three wild donors and the IR64 RP displayed positive phenol reaction, while Cybonnet seeds showed no discoloration. Based on this evidence, we determined that Cybonnet carried the non-functional *ph* allele, while IR64 carried the functional *Ph* allele, consistent with a majority of *Indica* varieties. We further confirmed this result by sequencing the *Ph* locus. Sequencing demonstrated that Cybonnet carried a 1-bp insertion in exon 1 (chr 4: 31,749,302 bp) causing a frameshift that rendered the protein non-functional. This was somewhat unexpected, as a well known 18 bp loss-of-function deletion in exon 3 is more characteristic of *japonica* varieties (Yu et al., 2008). Sequencing of the *BH4* gene confirmed that all three wild donor parents carried functional *BH4* alleles, while Cybonnet and IR64 both carried non-functional alleles caused by a common 22 bp deletion in exon 3 (Zhu et al., 2011). We next assessed hull color in the CSSLs and found that only three lines, all from the IR64 × OrB library, carried the black hull phenotype (individuals IR64_OrB_34, IR64_OrB_35, and IR64_OrB_81). Each of the black hulled lines contained a donor introgression at the *BH4* locus, but the introgression alone was not predictive of the phenotype; two lines from the same library carried donor introgressions at *BH4* (individuals IR64_OrB_15 and IR64_OrB_21), but did not have black hulls (Supplementary Table S2). These results suggest that if functional alleles at *Ph* and *BH4* alone were sufficient to cause black hull, any CSSL in the IR64 background (*Ph/bh4*) would need only a single introgression carrying a functional copy of *Bh4* to manifest the black hull phenotype, while a CSSL in the Cybonnet background (*ph/bh4*) would require an extended donor introgression covering both genes (∼8.8 Mb region). As summarized in Supplementary Table S2, we identified four CSSLs in the Cybonnet × OrA population carrying an introgression that potentially encompasses both *Ph* and *BH4*; however, these lines did not have black hulls, suggesting that none of them carried both genes. The paucity of markers in the region (Figure 4) precludes explicit definition of the introgression breakpoints. Sequencing the *Ph* and *BH4* regions of the five previously mentioned IR64 × OrB CSSLs potentially carrying the *BH4* introgression would further elucidate the mechanism underlying the black hull phenotype.

#### Shattering

Several major shattering genes have been identified in rice (Cai and Morishima, 2000; Onishi et al., 2007). *Shattering 4 (SH4)* is a member of the trihelix family of transcription factors and lies near the end of the long arm of chromosome 4, linked to the two major *black hull* loci described above (Li et al., 2006; Lin et al., 2007). *qSH1* is a BEL1-type homeobox-containing protein on chromosome 1 (Konishi et al., 2006). *OsShattering 1 (OsSh1)* is a YABBY transcription factor that was originally mapped as an ortholog of the *Shattering1* gene in sorghum (Lin et al., 2012); it is co-located with *qSH3*, a shattering QTL on chromosome 3 (Inoue et al., 2015; Ishikawa et al., 2017; Table 4 and Figure 4).

While shattering was not measured quantitatively in this study, 14 CSSLs were noted to have an unusually high degree of shattering such that they required bagging of panicles to ensure seed harvest (Supplementary Table S2). Thirteen of the 14 shattering CSSLs carried a donor introgression at *SH4* (Supplementary Table S2). The exception was CSSL “IR64_OrB_29” which had a shattering phenotype but did not carry donor alleles at *SH4*; however there was a donor introgression at the *OsSh1* locus on chromosome 3. Nine CSSLs from three different libraries (five from the IR64 × OrA library, two from the IR64 × OrC library, and two from the Cybonnet × OrC library) carried donor introgressions at *SH4* but were not observed to be shattering, and between 3 and 6 lines from each of the six libraries carried introgressions at *OsSh1* and/or *qSH1* with no obvious effect on the phenotype (Supplementary Table S2). We found no highly shattering phenotypes in the Cybonnet × OrA library, nor did we find any black hull phenotypes. This may be due to the fact that there was a paucity of SNP markers across the *BH4–SH4* region of chromosome 4 in this library, relative to all other libraries, which made it virtually impossible to select for the presence of donor introgressions in that region during CSSL development (Figure 4).

Seed shattering is the result of a developmentally programmed weakening or degradation of the abscission cell layer at the base of the grain where it attaches to the pedicel, and abscission layer formation is known to be regulated by multiple genes that control the timing and degree of seed shattering (Inoue et al., 2015). Large variation in the degree of seed shattering is observed in rice cultivars, with *Indica* varieties typically exhibiting higher degrees of shattering than *Japonica*. Consistent with this observation, 12 of the shattering lines in this study were from the IR64 libraries while only two were from the Cybonnet libraries. These results suggest that the major-effect genes described above interact with other genes in the genetic background to determine the extent and degree of shattering, and that the pattern of interacting background alleles in IR64 (*Indica*) differs from Cybonnet (*Japonica*). Thus, despite the presence of donor introgressions at *OsSh1/qSH3, qSH1* and *SH4* in many of our shattering CSSLs, it is not surprising that these loci, alone, are not predictive of the phenotype. These results open the door to further research using these materials to gain a deeper understanding of the genetic determinants of seed shattering in rice.

#### Flowering Time

Eight CSSLs in the Cybonnet background were observed to have modified phenology and displayed later-than-expected flowering under the long days of summer in the greenhouse in Ithaca, NY (12–16 h of daylight). Four of the lines derived from crosses with OrA, which itself had the earliest flowering of the three wild donors, and two lines each from the other Cybonnet libraries. Data on flowering time under long day conditions is not available for the IR64 libraries. Studies of flowering time in rice have demonstrated that a combination of large- and small-effect genes define the genetic architecture of the trait, and adaptation to a wide range of daylength and temperature regimes is mediated by complex gene × gene interactions in both wild and cultivated populations. To date, more than a dozen flowering time genes have been cloned and characterized (OGRO database)^6^ (Yamamoto et al., 2012) and numerous additional small-effect QTLs have been extensively documented (Bentley et al., 2013; Itoh and Izawa, 2013; Hori et al., 2015).

In this study we identified lines carrying donor introgressions at five major flowering time loci located on chromosomes 6, 7, and 10 (Figure 4; Table 4; and Supplementary Table S2). The genes belong to two independent signaling pathways, as summarized in Hori et al. (2015). The first pathway, referred to as the *OsGI-Hd1-Hd3a* (*rice GIGANTEA*, *Heading data 1*, *Heading date 3a*) pathway, corresponds to the *GI-CO-FT* (*GIGANTEA*, *CONSTANS*, *FLOWERING LOCUS T*) pathway in *Arabidopsis*. *Hd1* is a homolog of *Arabidopsis CO* and it promotes heading under short-day and represses it under long-day conditions (Yano et al., 2000). *HD1* promotes the expression of both *Hd3a* and its tandemly duplicated paralog, *Rice flowering locus T 1* (*RFT1*), under short-day conditions, but inhibits *Hd3a* and *RFT1* under long-day conditions (Kojima et al., 2002). *Hd3a* and *RFT1* function as florigens, or floral inducers (Tamaki et al., 2007; Ogiso-Tanaka et al., 2013). These three genes are all located on chromosome 6 in rice. The other pathway represents an independent signaling cascade that also regulates the florigens but is unique to rice. It includes *Early heading date 1* (*Ehd1*) and *Grain number, plant height and heading date 7* (*Ghd7*). *Ehd1* is expressed only under short-days and it promotes flowering by inducing transcription of *Hd3a* and *RFT1*, functioning independently of *Hd1* (Doi et al., 2004). *Ghd7* represses *Ehd1*, *Hd3a*, and *RFT1* under LD conditions, and does not affect *Hd1* mRNA levels (Xue et al., 2008). *Ehd1* is located on chromosome 10 and *Ghd7* on chromosome 7 (Figure 4).

Chromosome segment substitution lines containing introgressions at these five loci are summarized in Supplementary Table S2. For the four late-flowering lines from the Cyb × OrA library, we observed no relationship between the phenotype and the presence of donor introgressions at any of the above-mentioned flowering time loci. However, for the Cyb × OrB and Cyb × OrC libraries, the presence of a donor introgression at the *Hd1* locus was perfectly predictive of late flowering. In the case of Cyb_OrB_39, which was also late flowering, we see that the line carries an introgression across the *Hd3a* and *RFT1* genes, but does not include *Hd1*. This suggests that donor alleles at other genes are responsible for the phenotype in this line.

### Seed Production in CSSLs

During development of the CSSL libraries, we observed instances of low seed set (less than 200 seeds per plant under greenhouse or field conditions) in several lines per population (Supplementary Table S2). Overall, 28 lines were noted to have low levels of fertility (four in the Cyb × OrA, six in the Cyb × OrB, four in the Cyb × OrC, four in the IR64 × OrA, six in the IR64 × OrB, and four lines in the IR64 × OrC populations). Six lines in the Cybonnet × OrB and OrC libraries had low or no seed set in the field (Lafayette, LA, United States, 2019) but it was possible to obtain some seed set from the same plants grown under greenhouse conditions in Ithaca, NY, United States. Over the course of development, we also noticed that some lines were fertile in the heterozygous condition, but we were unable to harvest seed from homozygous offspring. In some of these cases, we were able to select sib lines that contained segment(s) of interest and produced seed when selfed, but over time, it became more difficult to find lines containing specific regions of introgression; in some cases, we lost segments entirely due to sterility (Supplementary Table S3). For example, in both the Cyb × OrB and IR64 × OrB libraries, a ∼650 kb region on chromosome 1 (41,985,829–42,630,129 bp) was only maintained as a heterozygous region and the seed set was always very low in the CSSLs carrying this introgression, indicative of the presence of a sterility gene(s). This region was implicated only in the OrB libraries, as sterility was not a problem in the corresponding lines developed from the OrA or the OrC donors. At this time, we have chosen to maintain some lines as backcross populations as an insurance policy to avoid losing additional segments. A case in point is Line Cyb_OrB_4 which contains six introgressions in chromosomes 1, 3, and 11, and is currently propagated by backcrossing to Cybonnet.

There are hundreds of loci known to contribute to varying levels of gametic and zygotic incompatibilities distributed throughout the 12 chromosomes of rice as noted in Oryzabase^7^ (Kurata and Yamazaki, 2006) and the OGRO database^6^; (Yamamoto et al., 2012). Most are population-specific, and this makes it difficult to associate the low fertility phenotypes with particular genes or introgressions in the CSSLs. Our objective in documenting CSSLs with low levels of seed set (as well as very late flowering) is to alert researchers which lines may be difficult to propagate and therefore require special attention. In some cases, we also alert users of these genetic resources that seeds of heterozygous backcross progeny or bulked seeds harvested from multiple generations of pure-line sibs may be distributed for particular lines.

### Functional SNPs on the C7AIR and Predicted Phenotypes of the CSSLs

The C7AIR includes eight functional SNPs in its design that target important agronomic or physiological traits (Morales et al., 2020). They tag causal variants in genes conferring blast disease resistance (*Pi-ta*; Bryan et al., 2000), grain size (*GS3*; Fan et al., 2006; Takano-Kai et al., 2009), and eating and cooking quality (*ALK*, *WAXY*; Okagaki and Wessler, 1988; Wang et al., 1995; Gao et al., 2003). The parents of all six libraries were polymorphic for these variants, and CSSLs carrying donor SNPs at *Pi-ta*, *GS3*, *ALK*, and *WAXY* were documented (Supplementary Table S2). At *GS3*, both Cybonnet and IR64 carried the allele for long grain, while the three wild parents carried the wild type allele conferring short-medium grain length. At *Pi-ta*, the two RPs carried the allele for resistance while the wild parents carried the ancestral *pi-ta* allele conferring blast susceptibility (Huang et al., 2008). The evolution of resistance at the *Pi-ta* locus is believed to have been the result of a mutation that coincided with domestication, so it is not entirely surprising that none of the wild donors used in this study were found to carry the resistant allele (Lee et al., 2009; Yoshida and Miyashita, 2009). We were unable to predict grain quality phenotypes in the CSSLs because the traits determined by *ALK* and *WAXY* are quantitative, epistatic, and post-transcriptionally regulated (Bligh et al., 1998; Tian et al., 2009; Huggins et al., 2019). Evaluating these lines for novel grain and nutritional quality attributes is an exciting area for future research.

### CSSL Identifier

To facilitate downstream use and evaluation of our six libraries by the greater rice genetics and breeding community, we developed a simple, user-friendly R Shiny Application that enables searching for lines of interest across user-selected libraries by inputting a genomic region of interest. The web application returns a comprehensive list of individual lines across the selected libraries that harbor introgressions either fully or partially across the region of interest from the donor accessions so that users may shortlist lines that they may potentially want to evaluate. Users may access the C7Air genotype data in Supplementary Dataset 2 or may also download C7AIR genotype data directly from the application for their filtered list of lines. This application is available at http://cssl-identifier.rcac.purdue.edu/ and is hosted by Purdue University Research Computing.

### Utility

The design and development of these CSSLs represents only the first step in an ambitious conceptualization of a public pre-breeding program that serves to develop, test, and deploy novel variation, with the long-term goal of enabling breeders to more readily tap into the hidden potential of wild and exotic plant genetic resources. The materials developed here are available for distribution and offer researchers new opportunities to undertake complementary multi-year, multi-location trials for yield and agronomic performance, response to abiotic and biotic stresses, and quality traits important to the rice community. To maximize the potential for wide utility and interpretation, phenotypes must be collected using controlled vocabularies, with accompanying climatic metadata and management details to provide environmental context. Further, experimental data describing phenotypic, genotypic and environmental variation should be deposited into a common database or data management system to make those data findable, accessible, interoperable and re-usable (Wilkinson et al., 2016) so that benefits can be collectively realized.

Pre-breeding is inevitably a long-term value proposition. Benefits will continue to accrue over many years if efforts are carried out in partnership with applied breeding programs dedicated to finished variety development. A partnership model that leverages public and private sector resources would enable the large scale experiments that are needed to drive recombination via rapid-cycle recurrent selection in order to overcome linkage drag and give rise to novel sources of useful variation. Favorable new materials could then be extracted by breeders periodically for crossing with elite lines, while the essential cauldron of diverse alleles were continuously managed, simmered and stirred. The potential of a well-designed, thoughtfully implemented pre-breeding program that is fueled by complementary partnerships, creative benefit-sharing agreements, and strategic deployment of resources has far-reaching consequences for plant breeders and for agricultural communities world-wide. Such a pre-breeding program can drive innovation, motivate new thinking, and unleash strategies that iteratively create value by reinvesting in the deployment of natural variation across the landscape, bringing rewards for decades to come.

## Data Availability Statement

The genotypic datasets presented in this study can be found in Supplementary Data 2—Genotype information (C7AIR) for CSSL populations. Sequencing data for grain and hull color genes from the wild and cultivated parents and selected CSSLs is available in GenBank with the following Accession IDs: *Bh4* (MW310629 - MW310643), *Rd* (MW310644 - MW310658), *Rc* (MW310659 - MW310674), *Ph* (MW310675 - MW310688).

## Author Contributions

NS, LA, HK, KA, SH, J-WK, ES, BM, S-NA, and GE contributed to the development of CSSL populations. NS, SH, GE, and HK phenotyped the plants. NS, KA, and SH genotyped the plants. YS and GD developed data management tools. VG and DW developed the CSSL Identifier R Shiny App. NS, DW, SH, GE, and SM analyzed the data and wrote the manuscript. SM conceptualized the project. All authors contributed to the article and approved the submitted version.

## Conflict of Interest

HK is currently employed by the company LG Chemical, Ltd. The remaining authors declare that the research was conducted in the absence of any commercial or financial relationships that could be construed as a potential conflict of interest.

## Footnotes

1 http://mapdisto.free.fr/CSSLFinder/

2 https://tassel.bitbucket.io/

3 https://www.R-project.org/

4 http://www.isbreeding.net/software/?type=detail&id=14

5 https://www.ars.usda.gov/GSOR

6 http://qtaro.abr.affrc.go.jp/ogro

7 https://shigen.nig.ac.jp/rice/oryzabase/
