# Corrigendum: A Coordinated Suite of Wild-Introgression Lines in *Indica* and *Japonica* Elite Backgrounds

**DOI:** 10.3389/fpls.2020.640122

**Published:** 2021-03-02

**Authors:** Namrata Singh, Diane R. Wang, Liakat Ali, HyunJung Kim, Kazi M. Akther, Sandra E. Harrington, Ju-Won Kang, Ehsan Shakiba, Yuxin Shi, Genevieve DeClerck, Byron Meadows, Vishnu Govindaraj, Sang-Nag Ahn, Georgia C. Eizenga, Susan R. McCouch

**Affiliations:** ^1^Plant Breeding and Genetics Section, School of Integrative Plant Science, Cornell University, Ithaca, NY, United States; ^2^Rice Research and Extension Center, University of Arkansas, Stuttgart, AR, United States; ^3^Department of Agronomy, Chungnam National University, Daejeon, South Korea; ^4^USDA-ARS Dale Bumpers National Rice Research Center, Stuttgart, AR, United States

**Keywords:** *Oryza sativa*, crop wild relatives, *Oryza rufipogon* Species Complex, chromosome segment substitution line, pre-breeding resources

In the original article, there was a mistake in **Data Availability Statement** as published. A correction has been made to **Data Availability Statement**. The corrected **Statement** appears below.

The genotypic datasets presented in this study can be found in Supplementary Data 2—Genotype information (C7AIR) for CSSL populations. Sequencing data for grain and hull color genes from the wild and cultivated parents and selected CSSLs is available in GenBank with the following Accession IDs: *Bh4* (MW310629 - MW310643), *Rd* (MW310644 - MW310658), *Rc* (MW310659 - MW310674), *Ph* (MW310675 - MW310688).

The authors apologize for this error and state that this does not change the scientific conclusions of the article in any way. The original article has been updated.

