# Supplementary material for: A Coordinated Suite of Wild-Introgression Lines in Indica and Japonica Elite Backgrounds
Source: Front Plant Sci. 2020 Nov 12;11:564824. doi: 10.3389/fpls.2020.564824 (PMC7688981; doi:10.3389/fpls.2020.564824)
Supplement: Supplementary file 1 [file Data_Sheet_1.docx]

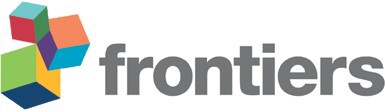


***Supplementary Material***

# Supplementary Data 1.

# Data Availability

# The genotypic datasets presented in this study can be found in Supplementary Data 2—Genotype information (C7AIR) for CSSL populations. Deposition of sequencing data for *Rc, Rd, BH1* and *BH4* is currently in process and GenBank Accession Numbers will be updated as soon as available.

# Genotyping methods employed during the development of six CSSL libraries.

Four genotyping platforms were utilized at different generations for foreground and background selection during CSSL development: Simple Sequence Repeat (SSR) (McCouch et al., 2002; Orjuela et al., 2010), 384-Oligo Pool Assay (OPA) (Thomson et al., 2012), the Cornell_6K_Array_Infinium_ Rice (C6AIR) (Thomson et al., 2012), and the C7AIR (Morales et al., 2020).

*SSR markers.* Each of the six crosses was genotyped using 10-15 SSR markers from the Universal Core Genetic Map (UCGM) to identify heterozygous F1 progeny (Orjuela et al., 2010). Polymerase chain reaction (PCR) was performed using 20-30ng of DNA template, primers, along with a premixed ready- to-use GoTaq® Green Master Mix (Promega). The PCR conditions and analysis of amplicons were as described by (Arbelaez et al., 2015). A single F1 plant for each library was used as the pollen donor for backcrossing with each RP.

*GoldenGate 384-Oligo Pool Assays (OPA).* Progenies of BC1- BC3 backcross generations were genotyped using two different OPAs (~1-2 SNPs/MB). One 384-OPA (RiceOPA5.0) was informative for the CSSLs developed in the IR64 (*Indica)* backgrounds, and the other (RiceOPA6.0) was informative for CSSLs developed in the Cybonnet (*Japonica)* background. A 2^nd^ generation 384-SNP OPA was later developed (RiceOPA5.1 and RiceOPA6.1) by replacing non-informative SNPs in the 1^st^ generation platform. RiceOPA5.1 called 317 informative SNPs for the IR64 CSSLs, and RiceOPA6.1 provided 333 informative markers for the Cybonnet CSSLs. Both OPAs were developed from SNPs on the 44,000-SNP Affymetrix custom-designed array (Tung et al., 2010; Zhao et al., 2011; Thomson et al., 2012). A DNA concentration of 150-250 ng/ul was used for fragmentation, hybridization, annealing, and base extension (Ilumina, Inc.). The products were fluorescently labeled and scanned through the Illumina BeadArray reader to generate intensity files, which were decoded on Genome Studio software.

*Illumina Infinium C6AIR and C7AIR platforms*. Subsequent generations (BC3-BC6) were genotyped using the C6AIR and C7AIR, which were designed to have a marker density of 4-6 SNPs/Mbp. These arrays are comprised of selected SNPs from both 384-OPAs, a SNP discovery panel which included all six CSSL parents, and the High Density Rice Array (HDRA) consisting of 700,000 SNPs (McCouch et al., 2016; Thomson et al., 2017; Morales et al., 2020). With the C6AIR and the C7AIR, a single array was capable of screening all six parental combinations and provided a resolution of 4-6 SNPs/MB. The number of informative markers for the 6 libraries varied between 1,000 and 2,500. The genotype information from the BC2-BC3 families run on 384-OPAs was easily tracked; donor and recurrent parent alleles were integrated into successive generations run on the C6AIR and C7AIR. The genotyping and SNP allele calling was carried out as described by Thomson et al. (2017) and Arbelaez et al. (2015). The percent heterozygosity and RP background for each line was calculated and lines to be advanced were selected based on presence of the target donor segment (foreground) and a high percent of RP background.

# REFERENCES

Arbelaez, J.D., Moreno, L.T., Singh, N., Tung, C.W., Maron, L.G., Ospina, Y., et al. (2015). Development and GBS-genotyping of introgression lines (ILs) using two wild species of rice, and in a common recurrent parent, cv. *Curinga*. *Mol Breed* 35(2)**,** 81. doi: 10.1007/s11032- 015-0276-7.

McCouch, S.R., Teytelman, L., Xu, Y., Lobos, K.B., Clare, K., Walton, M., et al. (2002).Development and mapping of 2240 new SSR markers for rice (*Oryza sativa* L.) (supplement). *DNA Res* 9(6)**,** 257-279.

McCouch, S.R., Wright, M.H., Tung, C.-W., Maron, L.G., McNally, K.L., Fitzgerald, M., et al. (2016). Open access resources for genome-wide association mapping in rice. *Nat Commun* 7. doi: 10.1038/ncomms10532.

Morales, K.Y., Singh, N., Perez, F.A., Ignacio, J.C., Thapa, R., Arbelaez, J.D., et al. (2020). An improved 7K SNP array, the C7AIR, provides a wealth of validated SNP markers for rice breeding and genetics studies. *PLoS One* 15(5), e0232479. doi: 10.1371/journal.pone.0232479.

Orjuela, J., Garavito, A., Bouniol, M., Arbelaez, J.D., Moreno, L., Kimball, J., et al. (2010). A universal core genetic map for rice. *Theor Appl Genet* 120(3)**,** 563-572. doi: 10.1007/s00122- 009-1176-1.

Thomson, M.J., Singh, N., Dwiyanti, M.S., Wang, D.R., Wright, M.H., Perez, F.A., et al. (2017). Large-scale deployment of a rice 6 K SNP array for genetics and breeding applications. *Rice (N Y)* 10(1)**,** 40. doi: 10.1186/s12284-017-0181-2.

Thomson, M.J., Zhao, K.Y., Wright, M., McNally, K.L., Rey, J., Tung, C.W., et al. (2012). High- throughput single nucleotide polymorphism genotyping for breeding applications in rice using the BeadXpress platform. *Molecular Breeding* 29(4)**,** 875-886. doi: 10.1007/s11032-011- 9663-x.

Tung, C.W., Zhao, K.Y., Wright, M.H., Ali, M.L., Jung, J., Kimball, J., et al. (2010). Development of a Research Platform for Dissecting Phenotype-Genotype Associations in Rice (*Oryza* spp.). *Rice* 3(4)**,** 205-217. doi: 10.1007/s12284-010-9056-5.

Zhao, K., Tung, C.-W., Eizenga, G.C., Wright, M.H., Ali, M.L., Price, A.H., et al. (2011). Genome- wide association mapping reveals a rich genetic architecture of complex traits in *Oryza sativa*. *Nat Commun* 2**,** 467. doi: 10.1038/ncomms1467
