# Supplementary material for: A Coordinated Suite of Wild-Introgression Lines in Indica and Japonica Elite Backgrounds
Source: Front Plant Sci. 2020 Nov 12;11:564824. doi: 10.3389/fpls.2020.564824 (PMC7688981; doi:10.3389/fpls.2020.564824)
Supplement: Supplementary file 3 [file Image_1.pdf]

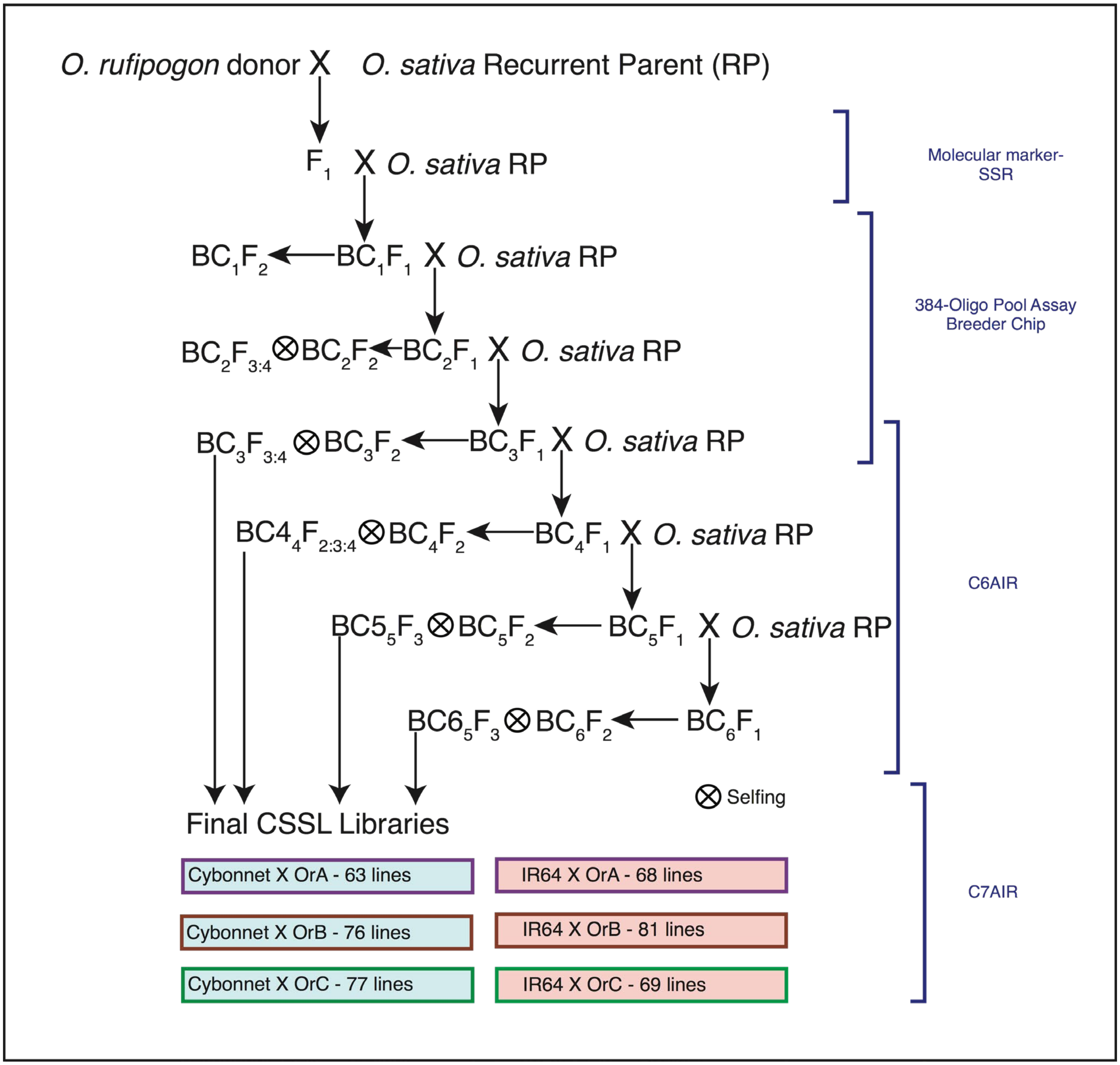

Supplementary Figure S1. Crossing scheme used to develop the six inter-specific CSSL libraries. Earlier generations were genotyped using Simple Sequence Repeat (SSR) markers, a 384-Oligo Pool Assay and the C6AIR and C7AIR SNP arrays. Final libraries were genotyped using the C7AIR SNP array.

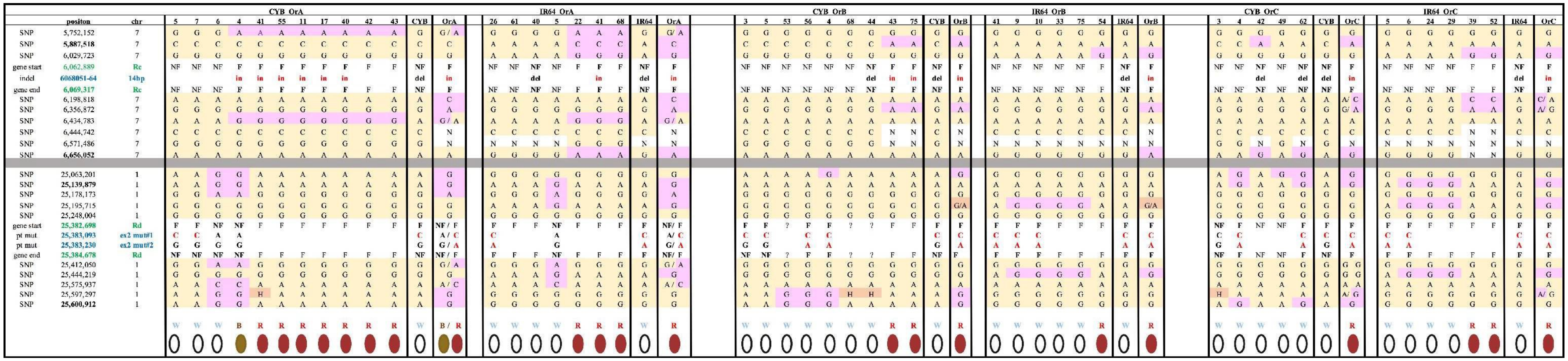

Supplementary Figure S2. Pericarp analyses on the six CSSL populations. In the *Rc* (chr. 7) and *Rd* (chr. 1) regions, the start and end positions of the *Rc* and *Rd* genes are identified in **green** text; known functional polymorphisms are in **blue** text; and SNPs from the C7AIR are in **black** text. Under each population name, the number and genotype of each CSSL shows which lines carried donor introgressions across the gene regions. Pericarp color is illustrated at the bottom as red (R), brown (B) and white (W). OrA was heterozygous at the *Rd* locus, thus segregated for red and brown pericarp. For each population, the parents are shown to the right. The alleles associated with red pericarp are in red text. The ORSC donor alleles are highlighted in purple, heterozygous alleles in dark brown, recurrent parent alleles in light brown and missing alleles in white. (Abbreviations: non-functional allele, NF; functional allele, F; insertion, in; deletion, del.)
